# Macrophage mediated recognition and clearance of *Borrelia burgdorferi* elicits MyD88-dependent and -independent phagosomal signals that contribute to phagocytosis and inflammation

**DOI:** 10.1186/s12865-021-00418-8

**Published:** 2021-05-17

**Authors:** Sarah J. Benjamin, Kelly L. Hawley, Paola Vera-Licona, Carson J. La Vake, Jorge L. Cervantes, Yijun Ruan, Justin D. Radolf, Juan C. Salazar

**Affiliations:** 1 Department of Pediatrics, UConn Health, Farmington, CT 06030 USA; 2 Department of Immunology, UConn Health, Farmington, CT 06030 USA; 3 Division of Infectious Diseases, Connecticut Children’s, Hartford, CT 06106 USA; 4 Center for Quantitative Medicine, UConn Health, Farmington, CT 06030 USA; 5 Department of Cell Biology, UConn Health, Farmington, CT 06030 USA; 6 Institute of Systems Genomics, UConn Health, Farmington, CT 06030 USA; 7 Present Address: Paul L. Foster School of Medicine, Texas Tech University Health Sciences Center, El Paso, TX 79905 USA; 8 The Jackson Laboratory for Genomic Medicine, Farmington, CT 06032 USA; 9 Department of Medicine, UConn Health, Farmington, CT 06030 USA; 10 Department of Molecular Biology and Biophysics, UConn Health, Farmington, CT 06030 USA; 11 Department of Genetics and Genomic Sciences, UConn Health, Farmington, CT 06030 USA; 12 Division of Pediatric Infectious Diseases and Immunology, Connecticut Children’s, 282 Washington Street, Hartford, CT 06106 USA

**Keywords:** Macrophage, Phagocytosis, Inflammation, Borrelia, MyD88

## Abstract

**Background:**

Macrophages play prominent roles in bacteria recognition and clearance, including *Borrelia burgdorferi* (*Bb*), the Lyme disease spirochete. To elucidate mechanisms by which MyD88/TLR signaling enhances clearance of *Bb* by macrophages, we studied wildtype (WT) and MyD88^−/−^ *Bb*-stimulated bone marrow-derived macrophages (BMDMs).

**Results:**

MyD88^−/−^ BMDMs exhibit impaired uptake of spirochetes but comparable maturation of phagosomes following internalization of spirochetes. RNA-sequencing of infected WT and MyD88^−/−^ BMDMs identified a large cohort of differentially expressed MyD88-dependent genes associated with re-organization of actin and cytoskeleton during phagocytosis along with several MyD88-independent chemokines involved in inflammatory cell recruitment. We computationally generated networks which identified several MyD88-dependent intermediate proteins (*Rhoq* and *Cyfip1*) that are known to mediate inflammation and phagocytosis respectively.

**Conclusion:**

Our findings show that MyD88 signaling enhances, but is not required, for bacterial uptake or phagosomal maturation and provide mechanistic insights into how MyD88-mediated phagosomal signaling enhances *Bb* uptake and clearance.

**Supplementary Information:**

The online version contains supplementary material available at 10.1186/s12865-021-00418-8.

## Background

Lyme disease (LD) is a highly prevalent tick-borne illness caused by the spirochetal bacterium *Borrelia burgdorferi* (*Bb*) [1–3]. The disease is characterized by a wide array of clinical manifestations which vary in duration and severity among patients. Early clinical manifestations of LD include the characteristic “bullseye” rash known as *erythema migrans* and flu-like symptoms, while late manifestations include arthritis, carditis and neurological compromise [4, 5]. The invading spirochete induces both innate and adaptive immune responses, and it is believed that the innate immune response to *Bb* contributes to the development of clinical findings in LD [6]. The macrophage is a principal cellular element of the innate immune response to the bacterium at sites of infection in both humans and mice [7–9]. Macrophages also play a prominent role in the pathogenesis of murine Lyme carditis, and their recruitment to heart tissue is important in spirochetal clearance [10]. Macrophages have the phagocytic and signaling machinery necessary to bind, engulf, and degrade *Bb*. Binding of *Bb* to macrophages is mediated by surface integrins, such as Complement Receptor 3 (CR3) [11, 12] and α_3_ [13]. Once attached, phagocytosis of *Bb* is complex and can occur by either a sinking or coiling mechanism [14, 15]. Both cases require rearrangements of the actin cytoskeleton to internalize Bb into the endosome, where degradation takes place [16].

*Bb* is an extracellular pathogen that needs to be taken up and degraded for significant recognition by the host immune system [17]. We have defined this process as “phagosomal signaling” [14]. Spirochete degradation exposes borrelial pathogen-associated molecular patterns (PAMPs), such as lipoproteins and nucleic acids, to endosomal toll-like receptors (TLRs) for recognition, resulting in signaling cascades which induce pro-inflammatory cytokine production [17–19]. *Bb* does not contain LPS and, therefore, does not engage TLR4. The cell envelope of *Bb* contains abundant triacylated lipoproteins [20], which are known to be recognized by TLR1/2 heterodimers [21–26]. However, the three fatty acid chains in the N-terminus of *Bb* lipoproteins, which serve as the TLR2/1 PAMP, are tethered in the outer membrane [27]. We have shown that this results in minimal recognition of lipoproteins in intact spirochetes at the cell surface [17, 18, 21, 28]. Instead, principal recognition of *Bb* TLR2 ligands occurs within macrophage endosomal structures after the spirochete is phagocytosed and degraded [17, 28]. Bacterial degradation results in exposure of both lipoprotein ligands and nucleic acids, which are recognized by endosomal TLR2 and TLRs 7, 8 and 9 respectively [18, 19, 29]. Signaling cascades initiated by engagement of these TLRs utilize the adaptor protein MyD88 [14, 30], indicating that this adaptor protein is a crucial element in mediating the inflammatory response to *Bb*.

A role for MyD88 has been implicated in each of the four general steps associated with phagocytic clearance of bacterial pathogens: uptake, phagosome maturation, degradation and cytokine production. Murine macrophages lacking MyD88 show markedly diminished uptake of several bacterial species, including *Bb* [28, 31–35]. In WT macrophages, prior studies have shown that *Bb*-induced MyD88 signaling results in increased PI3K activation and when PI3K is inhibited *Bb* uptake is decreased [36]. In addition, formin proteins (FMNL1, mDia1, and Daam1) have been shown to play a critical role in mediating phagocytosis of *Bb* [15, 37]. Whether MyD88 increases activation of these formins, and the role of PI3K signaling in this process, has not been established. Degradation of bacteria is impaired in the absence of MyD88 due to inefficient acidification of phagosomes [38]. In the context of *Bb* infection, lysosome maturation markers are recruited to *Bb*-containing phagosomes in macrophages lacking MyD88 [28]. However, the degree of phagosome maturation and acidification required to expose *Bb* ligands from the bacteria cell envelope for recognition has not been studied. Murine macrophages lacking MyD88 also show markedly diminished production of NFκB-triggered pro-inflammatory cytokines, such as TNFα and IL-6, when stimulated with different bacterial species, including *Bb* [28, 31]. Nevertheless, the key host components involved downstream of these MyD88-mediated phagosome signals and their effects have not been well studied in the context of *Bb* infection.

The objective of these studies is to examine which downstream effects of MyD88 phagosomal signaling potentially enhance clearance of *Bb.* Using an ex vivo murine macrophage system, we show that MyD88 signaling enhances, but is not required, for bacterial uptake or phagosomal maturation. Through RNA-sequencing analysis, we provide evidence that MyD88 signaling drives transcription of multiple genes involved in phagocytosis and identify potential intermediate proteins that facilitate the association between MyD88 and bacterial uptake. We also demonstrate that internalization of *Bb* by macrophages induces robust MyD88-independent inflammatory responses via production of chemokines. Our findings highlight the importance of MyD88 in efficient uptake of the Lyme disease spirochete by macrophages and provide potential mechanistic insight into how MyD88 mediates this process.

## Methods

### Mice

Female 6–8-week-old C57BL/6 J wild type (WT) and C57BL/6 J MyD88^−/−^ (MyD88^−/−^) mice used in these studies were obtained from breeding colonies maintained in the UConn Health (UCH) Center for Comparative Medicine facility according to guidelines set by the UCH Institutional Animal Care and Use Committee (IACUC). Female mice were used exclusively in these studies due to differences in expression of TLR7 between males and females [39]. Original WT breeding pairs were purchased from The Jackson Laboratory. Original MyD88^−/−^ breeding pairs were kindly provided by Dr. Egil Lien at the University of Massachusetts with permission from Dr. S. Akira in Osaka, Japan. Disruption of the murine MyD88 gene was confirmed through PCR [40]. Both WT and MyD88^−/−^ breeding colonies are maintained on the antibiotic Sulfatrim (sulfomethoxazole [40 mg/mL] + trimethoprim [8 mg/mL]) diluted in water 1:50, which has been previously shown to not impact the degree of *Bb* infection [41]. In preparation for euthanasia, individual mice were exposed to isoflurane to calm the animal prior to sedation by injection with an overdose of an anesthetic cocktail [Ketamine 50–75 mg/kg at 15 mg/ml, Xylazine 5–7.5 mg/kg at 2.5 mg/ml, and Acepromazine 0.5–1.25 mg/kg]. Following euthanasia by cervical dislocation, each animal was confirmed to lack a heartbeat in accordance with the approved IACUC protocol.

### Bacterial strains

Low-passage virulent wild-type *Bb* strain 297 [42] or a strain 297 isolate containing a stably-inserted copy of green fluorescent protein (GFP) under the control of the constitutively-expressed *flaB* promoter (Bb914) [43] were maintained in Barbour-Stonner-Kelly (BSK)-II media supplemented with normal rabbit serum and gentamicin (50 μg/μl) [43]. Cultures were grown at 23 °C for at least 1 week prior to being shifted to 37 °C as previously described [43]. Spirochetes were centrifuged at 3300 x *g* for 20 min at 4 °C and resuspended in either BSK-II for in vivo experiments or DMEM (Gibco, 15,630–080) supplemented with sodium pyruvate (Gibco, 11,360–070) and HEPES (Gibco, 15,630–080) for ex vivo experiments. After resuspension, the cultures were counted by dark-field microscopy using a Petroff-Hausser counting chamber (Hausser Scientific) and diluted accordingly. *Staphylococcus aureus* (*Sa*) was cultured and fluorescently labeled with FITC as previously described [44].

### BMDM stimulation

Bone marrow-derived macrophages (BMDMs) were isolated from 6 to 8-week-old WT and MyD88^−/−^ mice as described previously [18]. Single cell macrophage suspensions were seeded into either 12-well tissue culture-treated plates at a concentration of 1 × 10^6^ cells/ml per well or 1 × 10^5^ cells/500 μL per well in 8-chamber cell microscopy slides. Cells were then incubated overnight at 37 °C/5% CO_2_ to allow cell adherence before experimentation. Cells were incubated for either 0.5, 1, 4 or 6 h at 37 °C/5% CO_2_ with live GFP-*Bb* or labeled *Sa* at multiplicities of infection (MOIs) of either 10 or 100. Stimulation media was DMEM supplemented with 1% sodium pyruvate and 1% HEPES. At the end of the incubation period, culture supernatants were collected and stored at − 80 °C until cytokine analysis. Cells stimulated in chamber slides were processed for confocal microscopy. Cells stimulated in 12-well plates were processed for RNA extraction. All culture media and reagents were confirmed free of LPS contamination (< 10 pg/ml) by Limulus amoebocyte lysate assay quantification (Cambrex, MA).

### Confocal microscopy

After stimulation, BMDMs were fixed in 2% paraformaldehyde with 0.05% Triton-X-100 (Fisher, BP151–100) for 10 min. Slide wells were then incubated with 5% bovine serum albumin (BSA) solution in PBS overnight at 4 °C to block non-specific antibody binding. The next day, cells were stained with different combinations of anti-GFP (Thermo Scientific A-21311, 1:100), phalloidin conjugated with Alexa Fluor 647 (Biolegend 424205, 1:20), anti-MyD88 (Santa Cruz 11356, 1:100), anti-TLR2 (eBioscience 14–9021-82, 1:100), anti-TLR7 (R&D MAB7156, 1:100), anti-ASC (Santa Cruz 22514-R, 1:100) and anti-LAMP-1 (eBioscience 14–1071-82, 1:100). A secondary antibody, Alexa Fluor 350, was used to detect anti-MyD88, anti-TLR2, anti-ASC and anti-LAMP-1 (Life Technologies A21093, 1:100). Incubations with primary and secondary antibodies were done for 1 h each at room temperature; slide wells were washed following each incubation three times with PBS supplemented with 0.5% Tween-20, with a final wash in distilled H_2_O before mounting. After antibody staining, slides were mounted using Vectashield (Vector H-1000) and imaged using a Zeiss 880 confocal microscope. Image processing and analysis were performed using ImageJ (NIH, v1.41b). Colocalization values were determined by first analyzing profile plots in ImageJ (Plug-in: “Plot Profile”) across ten different phagosomes for each cell genotype and then calculating the average difference between the fluorescence intensity curves of the markers of interest (i.e., LAMP-1 and *Bb*). Binding percentages were calculated by imaging 100–200 cells using a confocal microscope and then measuring the ratio of cells containing at least one surface-bound or internalized spirochete to the total number of cells imaged for each condition, represented as %BMDMs interacting w/*Bb*. Uptake percentages were calculated by imaging 100–200 cells using a confocal microscope and then measuring the ratio of cells containing at least one internalized spirochete to the total number of cells imaged for each condition, represented as %BMDMs w/internalized *Bb*.

### Western blotting of BMDM supernatants and lysates

Protein lysates were generated from BMDM cell culture lysates and supernatants after *Bb* stimulation. In these experiments, adenosine triphosphate (ATP) (Sigma, 3A6419-1G) was added to WT BMDMs (already stimulated with *Bb* for 5 h) 1 h prior to harvest for generation of lysates. Supernatants were treated with an equal volume of methanol and ¼ volume of chloroform, vortexed and spun at 16000 x *g* for 10 min. After removal of the upper phase, 500 μL of methanol was added to the intermediate phase, which was then vortexed and spun at 16000 x *g* for 10 min. The pellets were then dried at room temperature, resuspended in 30 μL of 2x Laemmli buffer and incubated in a 37 °C water bath until proteins became soluble. BMDMs were lysed using RIPA buffer at − 80 °C and spun at maximum speed for 10 min. Protein pellets were resuspended in 2x Laemmli buffer. Lysates were boiled at 99 °C for 10 min and run on a 12.5% SDS-PAGE gel at 140 V for 1 h (5 μL per lane, 15 lanes). Proteins were then transferred to nitrocellulose membranes (Bio-Rad 162–0177) at 20 V for 20 min. Membranes were blocked for 1 h in milk block solution and then incubated overnight at 4 °C with primary antibodies for either β-actin (Sigma A5441, 1:2000), IL-1β (R&D AF401NA, 1:800) or caspase-1 (Adipogen AG-20B-0042, 1:1000) diluted in milk block solution. Membranes were then washed 5 times for 5 min each in wash buffer (PBS supplemented with 0.5% Tween-20) and incubated with goat anti-mouse HRP-conjugated IgG (GE NA931) diluted 1:5000 (β-actin and Caspase-1) or 1:1000 (IL-1β) in milk block for 2 h at room temperature. Following additional washes, membranes were incubated in HyGlo spray chemilunescent substrates (Denville Scientific, E2400) for 5 min and imaged on a Biorad ChemiDoc MP imaging system.

### Cytokine analysis

The Cytokine Bead Array Mouse Inflammation kit (BD Biosciences 552364) was used according to manufacturer’s instructions for simultaneous measurement of IL-6, IL-10, CCL2, IFNγ, TNFα, and IL-12p70 in supernatants from stimulated BMDMs. General statistical analysis was performed using GraphPad Prism 4.0 (GraphPad Software, San Diego, CA), using an unpaired Student *t* test. For each experiment, both the standard deviation and the standard error of the mean were calculated. *P*-values of < 0.05 were considered significant.

### Identification of differentially expressed genes by RNA-Seq

Total RNA was extracted from three biological replicates of WT and MyD88^−/−^ BMDMs, either unstimulated or stimulated with *Bb* at MOI 10:1 or MOI 100:1 for 6 h. Following stimulation, RNA was isolated using the Macherey-Nagel total RNA isolation kit (Takara, 740955) and was used as input for the NuGen Ovation RNA-seq V1 kit. cDNA output was analyzed for correct size distribution with an Experion Standard Sensitivity RNA chip and quantified using a Qubit Fluorometer. Sequencing libraries were produced using the NuGen Encore NGS Library I kit. Libraries were multiplexed and sequenced at The Jackson Laboratory for Genomic Medicine Sequencing Core with an Illumina HiSeq 2500 as 2X50bp pair end reads. RNA-Seq reads from each individual library were mapped with Tophat2 RNA-Seq spliced reads mapper (version 2.0.5) [45] to mouse genome build mm9 with parameter settings adjusted to suit strand-specific pair-end RNA-Seq reads. The mapping result bam files were used as input to the HTSeq high-throughput sequencing data analysis package [46] to quantify the read counts mapped to all genes in UCSC mm9 mouse gene annotation set. The expression levels of genes represented as mapped read counts were normalized using the DESeq2 RNA-Seq analysis package (function: *estimateSizeFactor*) [47]. Genes were considered expressed if the number of reads was above the 25th percentile for the normalized data set. For quality control, only replicates with Pearson correlation coefficient above 0.9 on their FPKM values were considered (Figure S1). Expressed genes were then further analyzed for differential gene expression using the DEseq2 package with FDR cutoff: 0.1. Differential gene expression was calculated in WT BMDMs stimulated 10:1 with *Bb* relative to unstimulated WT BMDMs and MyD88^−/−^ BMDMs stimulated 100:1 with *Bb* relative to unstimulated MyD88^−/−^ BMDMs. Differentially expressed genes (DEGs) were classified as either up-regulated or down-regulated based on the log2 of the fold change compared to the unstimulated control, which was calculated in R statistical software using package “DESeq2”. Determined DEGs were then separated into five groups based on their expression profiles; WT (all DEGs in WT BMDMs), MyD88^−/−^ (all DEGs in MyD88^−/−^ BMDMs), MyD88-dependent (all DEGs in WT but not MyD88^−/−^ BMDMs), MyD88-independent (all DEGs in both WT and MyD88^−/−^ BMDMs), and MyD88-privative (all DEGs in MyD88^−/−^ but not in WT BMDMs).

### Identification of enriched transcription binding sites and master regulator analysis

Transcription factor binding sites in promoters of differentially-expressed genes were analyzed using known DNA-binding motifs described in the TRANSFAC library [48], release 2017.2, available in the GeneXplain software (http://genexplain.com). Binding site enrichment analysis for each one of our sets of DEGs was carried out as part of a GeneXplain dedicated workflow. The background consisted of 300 mouse house-keeping genes and the TRANSFAC mouse Positional Weight Matrices PWM (motifs) for binding site prediction with *p*-value < 0.001 score cutoff. Promoters were extracted by the workflow with a length of 600 bp (− 500 to + 100) and an enrichment fold of 1.0.

Master regulatory molecules were searched for in signal transduction pathways upstream of the identified transcription factors. The GeneXplain workflow available for this analysis was used in conjunction with the GeneWays database. Parameters set included a maximum radius of 10 steps upstream of the transcription factor nodes, the DEG lists from the respective group as context genes and a z-score cutoff of 1.0. All transcription factors and master regulators used in the network analysis had confirmed expression in respective conditions using the total gene expression lists from the RNA-sequencing data set.

### Gene ontology (GO) enrichment analysis

A Gene Ontology (GO) enrichment analysis was performed for the different sets of DEGs, transcription factors, and master regulators using the TRANSPATH [49] database through GeneXplain software. Input sets were the DEGs, transcription factors, or master regulators from either the MyD88-dependent, MyD88-independent, or MyD88-privative groups. Focus was directed to the GO biological processes output. GO biological processes related to *Bb* uptake, inflammation, and chemotaxis were identified by first reviewing previous studies for any genes involved in response to *Bb* relating to these phenotypes. Enrichment analysis was performed on these genes to identify GO biological processes that hit at least 60% of the genes on the list, generating a list of relevant GO biological processes. Then an intersection was performed between the list of GO biological processes identified using our DEG, transcription factor, or master regulator lists, and the GO biological processes identified from the relevant genes. Heat maps of expressed genes hits in each biological process were done in R statistical software using package “ggplots”.

### Network reconstruction and network analysis

Networks were constructed joining the three identified layers on the networks: DEGs, transcription factors, and master regulators. The subnetworks were extracted from identified master regulators of interest. From the MyD88-dependent master regulator group, effort was directed on linking MyD88 with transcription factors that had binding sites in the promoter regions of the MyD88-dependent DEGs enriched in uptake biological processes. These transcription factors were identified using the TRANSPATH database with the enriched DEGs of interest as input. The output list of transcription factors was intersected with the list of transcription factors that were only expressed in WT BMDMs. Networks were assembled and analyzed using Cytoscape software [50]. To extract the desired subnetworks, we used OCSANA [51] within the BiNOM plugin [52] in Cytoscape 2.8.3. MyD88 was considered as a source node and transcription factors from the intersected list as target nodes. For MyD88-privative chemotaxis subnetwork construction the same analysis pipeline was applied. MyD88-privative master regulators significantly enriched in chemotaxis were used as source nodes and MyD88-privative transcription factors enriched in chemotaxis were used as targets.

## Results

### MyD88-deficient macrophages show comparable binding but reduced uptake of *Bb*

**Fig. 1:**
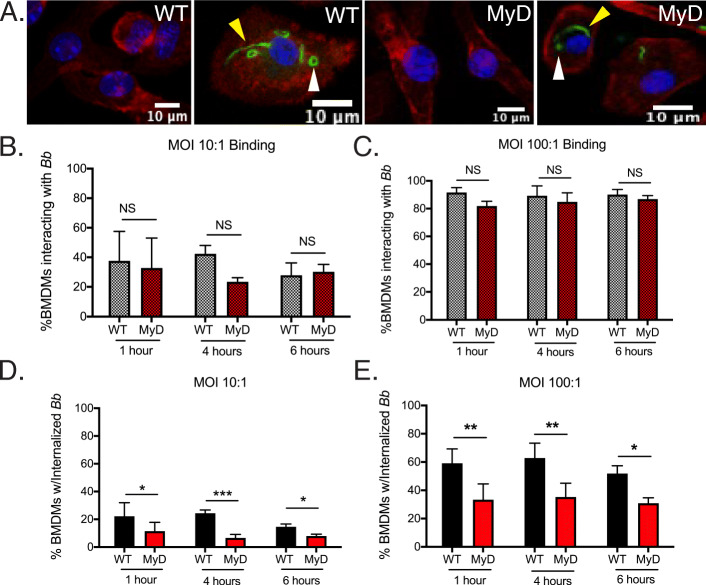
Quantitation of *Bb* binding and uptake by WT and MyD88^−/−^ BMDMs. **a** Confocal 40x images of WT and MyD88^−/−^ BMDMs after 6 h of stimulation with *Bb* at MOI 10:1, highlighting bound (yellow arrows) and internalized (white arrows) spirochetes. Green is *Bb*, red is actin and blue is cell nucleus. **b-c** Quantitation of bound spirochetes to WT (grey bars) or MyD88^−/−^ (dark red bars) BMDMs after 1, 4 or 6 h of stimulation at a MOI of 10:1 (**b**) or 100:1 (**c**). **d-e** Quantitation of internalized spirochetes to WT (black bars) or MyD88^−/−^ (red bars) BMDMs after 1, 4 or 6 h of stimulation at MOI 10:1 (**d**) or 100:1 (**e**). *n* = 3–5 mouse BMDM experiments per genotype *p-value< 0.05, **p-value< 0.01, ***p-value< 0.001, NS = not significant

The macrophage is an essential cellular element of the human inflammatory response to the LD spirochete [7]. Macrophages have also been shown as part of the inflammatory cell infiltrate in heart and joint tissue of mice experimentally infected with *Bb* [10, 53], and the importance of MyD88 in *Bb* clearance from mouse tissues has been previously reported [41, 54, 55]. It has also been well established that MyD88 enhances phagocytosis of multiple bacterial species by macrophages [28, 32, 34, 35, 56]. To better understand the contribution of MyD88 to spirochete binding, uptake and degradation by macrophages, we utilized an ex vivo macrophage model using WT and MyD88^−/−^ BMDMs co-incubated with *Bb* at MOIs of either 10:1 or 100:1 for 1, 4 or 6 h. To quantify binding percentages, we imaged macrophages by confocal microscopy and determined the number of cells with spirochetes either attached to the surface or internalized because internalized spirochetes had to bind to macrophages before being taken up (Fig. 1a, yellow and white arrows respectively). We used the same confocal images and total cell numbers to quantify uptake percentages based on the number of cells with internalized spirochetes. The percentages of cells with spirochetes either bound or internalized were comparable between WT and MyD88^−/−^ BMDMs at all three time points irrespective of MOI (Fig. 1b and c). While macrophages of both genotypes were able to phagocytose *Bb*, MyD88^−/−^ BMDMs showed significantly reduced spirochete uptake compared to WT BMDMs at MOI 10:1 (Fig. 1d). Increasing the MOI to 100:1 significantly enhanced uptake in both cell genotypes, but MyD88^−/−^ BMDMs never reached the phagocytic potential of their WT counterparts (Fig. 1e). These results further support the necessity of MyD88 signaling for efficient phagocytosis of *Bb*, irrespective of contact time with the spirochete.

### TLR2, TLR7 and MyD88 are recruited to *Bb*-containing phagosomes in macrophages

**Fig. 2:**
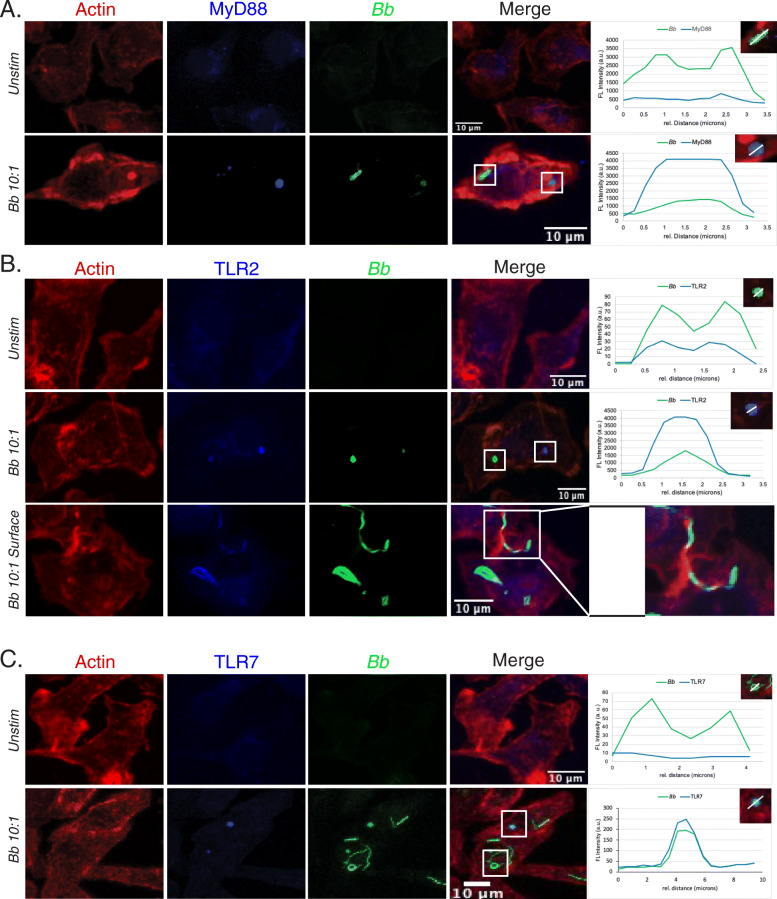
MyD88, TLR2 and TLR7 colocalize with *Bb* in phagosomes. **a-c** Confocal 40x images and colocalization analysis of internalized *Bb* with MyD88 (**a**), TLR2 (**b**) or TLR7 (**c**) in WT BMDMs after stimulation at MOI 10:1. White box indicates phagosome depicted in inset. Large inset in (**b**) shows coiling pseudopod formation around *Bb* on cell surface. Graph shows the intensity of each indicated pixel marker across the white line (distance on x-axis). Green is *Bb*, blue is MyD88 (**a**), TLR2 (**b**) or TLR7 [7], and red is actin

Once spirochetes are phagocytosed by macrophages, recruitment of TLR and MyD88 proteins to the phagosome is essential to trigger MyD88-dependent signaling cascades [57–60]. Importantly, we have demonstrated that in human monocytes TLR2 and TLR8 co-localize to endosomes containing *Bb* [19]. In addition, other investigators have shown a prominent role for TLR7 in the *Bb* inflammatory response [61]. Murine TLR8, unlike murine TLR7 and human TLR8, does not seem to utilize ssRNA as its ligand [62]. We therefore next characterized co-localization of TLR2, TLR7 and MyD88 with phagosomes containing *Bb* in BMDMs. By confocal microscopy, we observed that in WT BMDMs there is colocalization of MyD88 (Fig. 2a), TLR2 (Fig. 2b) and TLR7 (Fig. 2c) with *Bb*-containing phagosomes. Signals from MyD88 and TLR2 distinctly overlap with *Bb* GFP signals from phagosomes showing evidence of coiled or degraded spirochetes (Fig. 2a and b, graphs), but the intensity of MyD88 or TLR2 signal observed was higher with phagosomes containing degraded spirochetes. We also noted that TLR2 was expressed on the cell membrane and showed colocalization with surface-bound spirochetes (Fig. 2b). The absence of fluorescence in controls with secondary antibody only confirmed that this colocalization was not due to spectral overlap between color channels (Figure S2). TLR7 only showed strong signal with phagosomes containing partially degraded *Bb* but did not colocalize with surface-bound or recently internalized spirochetes (Fig. 2c). Taken together, these data confirm that endosomal TLR2, TLR7 and MyD88 colocalize to *Bb*-containing phagosomes to facilitate recognition of bacterial ligands and early response to infection.

### Lack of MyD88 does not affect degradation of *Bb* in the phagosome

**Fig. 3:**
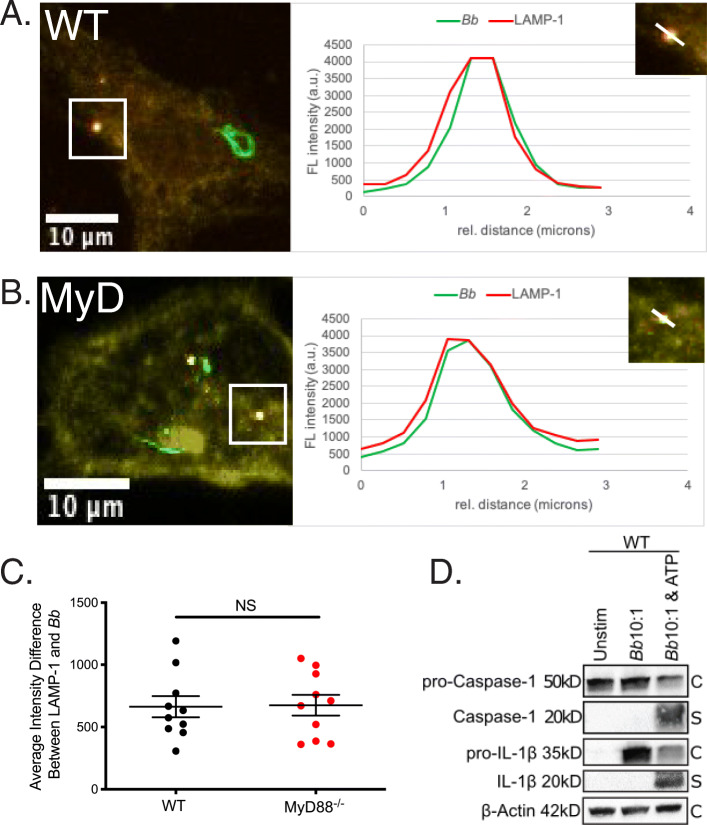
Colocalization of phagosome markers with internalized Bb in WT and MyD88−/− BMDMs. **a-b** Confocal 40x images of WT (**a**) and MyD88−/− (**b**) BMDMs after 6 h stimulation with Bb at MOI 10:1, depicting colocalization of Bb-containing phagosomes with LAMP-1. White box indicates phagosome depicted in inset. Graph shows the intensity of each indicated pixel across the white line (distance on x-axis). Green is Bb, red is LAMP-1 and yellow is actin. **c** Quantitation of colocalization between Bb and LAMP-1 in 10 phagosomes of WT (black dots) and MyD88−/− (red dots) BMDMs by measuring intensity difference between LAMP-1 staining and Bb staining. **d** Western blot of protein lysate isolated from WT BMDMs after 6 h stimulation with Bb +/− ATP (C = cell lysate, S = supernatant)

Degradation of the spirochete in the phagosome is crucial to expose bacterial ligands for recognition by endosomal TLRs [17]. This process, known as phagosome maturation, requires reduction of phagosome pH and fusion with lysosomes [63]. Given that both WT and MyD88^−/−^ BMDMs bind and internalize *Bb*, we next sought to determine if spirochetes are similarly degraded in phagosomes with and without MyD88. Confocal images taken after a 6-h stimulation at MOI 10:1 showed that both WT and MyD88^−/−^ BMDMs contained degraded GFP+ *Bb* within the cell actin matrix (Fig. 3a and b). To assess phagosome maturation, we quantitated recruitment of LAMP-1 to *Bb*-containing phagosomes by looking at colocalization of LAMP-1 and GFP fluorescence intensity [64]. Both WT and MyD88^−/−^ BMDMs showed comparable LAMP-1 and *Bb* colocalization in phagosomes (Fig. 3a and b, graphs). Colocalization between *Bb* and LAMP-1 was measured in multiple phagosomes in BMDMs from both genotypes and no significant differences were found (Fig. 3c). To confirm MyD88 signaling in response to *Bb* we also measured cytokine secretion after 1, 4 and 6 h of incubation with spirochetes. WT BMDMs showed significant increase in IL-6, TNFα and IL-10 secretion in the presence of spirochetes, whereas MyD88^−/−^ BMDMs did not (Figure S3A-C). Consistent with prior studies by Behera et al. (2006), both WT and MyD88^−/−^ BMDMs secrete the macrophage chemokine CCL2 (Figure S3D).

### *Bb* ligand recognition appears to occur solely from within the phagosome

To test for the presence of bacterial products in the cytosol, we measured cleaved caspase-1, which is indicative of inflammasome activation. Western blot analysis of WT BMDM cell lysates and supernatants showed no activation of caspase-1 by stimulation of *Bb* alone (Fig. 3d), which is consistent with previously published studies [65]. However, in discordance with previous studies [66], we did not see cleavage of IL-1β (Fig. 3d) unless exogenous ATP was added to the stimulation. To further confirm lack of NLRP3 inflammasome activation, we assessed Apoptosis-associated speck-like protein containing a CARD (ASC) in BMDMs stimulated with either *Bb* or *Staphylococcus aureus* (*Sa*) for 30 min or 6 h (Figure S4). As previously reported [67] (Figure S4A and C), ASC activation was observed with *Sa*, but no ASC was observed in BMDMs stimulated with *Bb* at 30 min or 6 h (Figure S4B and D). Thus, recognition of *Bb* ligands appears to occur solely within the phagosome.

### MyD88-dependent signaling causes differential expression of genes in macrophages that promote the inflammatory response

**Fig. 4:**
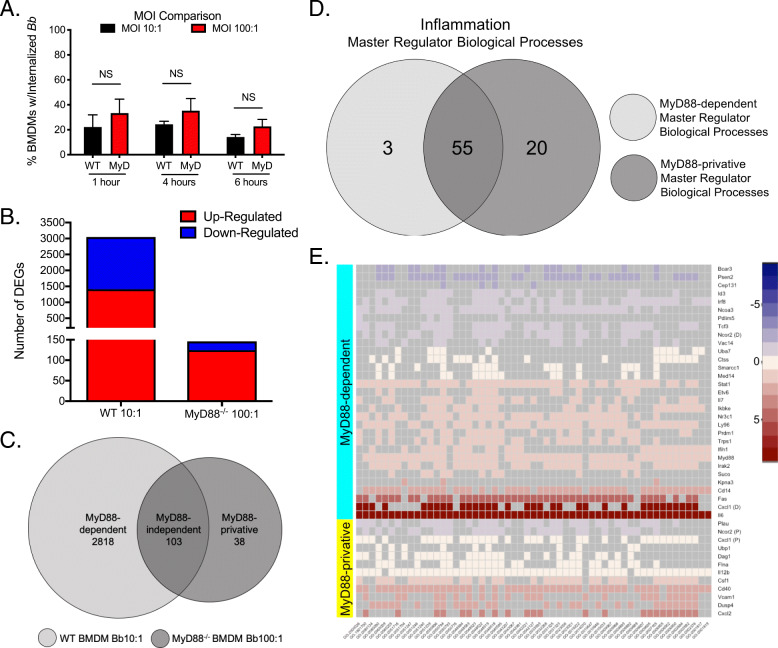
MyD88-dependent and independent MRs are significantly enriched in biological processes related to inflammation and chemotaxis. **a** Comparison of *Bb* internalization by WT BMDMs (black bars) at MOI 10:1 with MyD88^−/−^ BMDMs (red bars) at MOI 100:1. **b** Number of differentially expressed genes (DEGs) in *Bb*-infected WT and MyD88^−/−^ BMDMs determined by RNA-sequencing. Red bar indicates number of upregulated DEGs and blue bar indicates number of down-regulated DEGs. Bar height represents total number of DEGs in each condition. **c** Venn diagram depicting DEG classification. MyD88-dependent genes (light gray, left) are only differentially expressed in WT BMDMs. MyD88-independent genes (center) are expressed in both cell types. MyD88-privative genes (dark gray, right) are only differentially expressed in MyD88^−/−^ BMDMs. **d** Venn diagram comparing biological processes (BP) relating to inflammation significantly enriched between MyD88-dependent (light gray) and MyD88-privative (dark gray) master regulators. **e** Heat map showing fold change of master regulators enriched in inflammation in WT (cyan) or MyD88^−/−^ (yellow) BMDMs. GO numbers for significantly enriched BP are indicated on the x-axis. Heat maps were generated using R statistical software, package “heatmap2”

Our results above show that MyD88 expression in macrophages enhances their capacity to phagocytose spirochetes (Fig. 1). To gain a better understanding of events that occur downstream of signaling by MyD88 which result in this phenotype presentation, we performed RNA-sequencing on WT and MyD88^−/−^ BMDMs stimulated with *Bb* for 6 h. This time point was selected based on our data in Fig. 3 showing comparable maturation in both WT and MyD88^−/−^ BMDM phagosomes. We sequenced RNA from WT BMDMs at a MOI of 10:1 and MyD88^−/−^ BMDMs at a MOI of 100:1 for a comparative analysis because the uptake percentages were not significantly different between the two cell phenotypes under these conditions (Fig. 4a). Both WT and MyD88^−/−^ BMDMs showed differentially expressed genes (DEGs) when compared to their respective unstimulated controls. We noted that the number of DEGs in WT BMDMs was much higher than in MyD88^−/−^ BMDMs (2818 genes vs 141 genes respectively) (Fig. 4b). We saw similar numbers of up- and down-regulated DEGs in WT BMDMs (52 and 48%) (Fig. 4b). In the MyD88^−/−^ BMDMs, approximately 83% of the DEGs were up-regulated (Fig. 4b). We classified the DEGs into three categories for further analysis: genes differentially expressed only in WT BMDMs (MyD88-dependent); genes differentially expressed in both WT and MyD88^−/−^ BMDMs (MyD88-independent); and genes that were differentially expressed only in MyD88^−/−^ BMDMs (MyD88-privative) (Fig. 4c).

### Similar inflammatory and chemotactic processes are enriched regardless of MyD88-mediated signaling but utilize different regulatory proteins

MyD88-dependent mechanisms of inflammation have been well characterized, but little work has been done to understand the drivers of *Bb*-induced inflammation in the absence of MyD88. To address this issue, we next completed a comprehensive bioinformatics analysis to gain insight into how the DEGs are regulated within *Bb*-infected macrophages, both in the presence or absence of MyD88. We first identified transcription factors with potential binding sites in the promoter regions of the DEGs for each of the three subsets (66 for MyD88-dependent, 201 for MyD88-independent, and 39 for MyD88-privative). We then identified master regulator proteins upstream of these transcription factors and performed a Gene Ontology (GO) enrichment analysis of each group. Because data shown in Fig. 1 and Figure S3 indicate that in macrophages MyD88 affects both the inflammatory response and uptake of spirochetes, we focused our analysis on identifying whether any master regulators enriched to inflammatory and/or phagocytic biological processes in the MyD88-dependent and -privative conditions. Interestingly, similar inflammatory biological processes enriched to both the MyD88-dependent (including *MyD88*, *Irak2* and *Ly96*) and MyD88-privative (including *Vcam1* and *Cxcl2*) master regulators (Fig. 4d and e), but the individual master regulators involved were different for each subset (Fig. 4e). Importantly, over three times as many master regulators were identified for the MyD88-dependent DEGs than the MyD88-privative DEGs (Fig. 4e), suggesting that MyD88 signaling controls activation of more master regulators in the cell to control expression DEGs and enables the cell to perform unique processes in response to bacterial pathogens such as *Bb*.

### MyD88-privative master regulators are involved in multiple chemotactic biological processes not enriched in WT BMDMs

We also observed significant overlap between the chemotactic biological processes enriched in MyD88-dependent and MyD88-privative master regulators. However, MyD88-privative master regulators significantly enriched to multiple biological processes involved in chemotaxis that were not enriched in MyD88-dependent master regulators (Figure S5A), suggesting that the lack of MyD88 signaling allows for increased up-regulation of processes to facilitate cell migration into the tissues. The MyD88-privative master regulators involved in these chemotactic processes also enriched to inflammatory processes (Figure S5B and Fig. 4e), suggesting that *Bb* may trigger other signaling cascades which induce inflammation more skewed to cell recruitment and localization.

### MyD88 is a master regulator for transcription factors that control the MyD88-dependent DEGs enriched in uptake processes

**Fig. 5:**
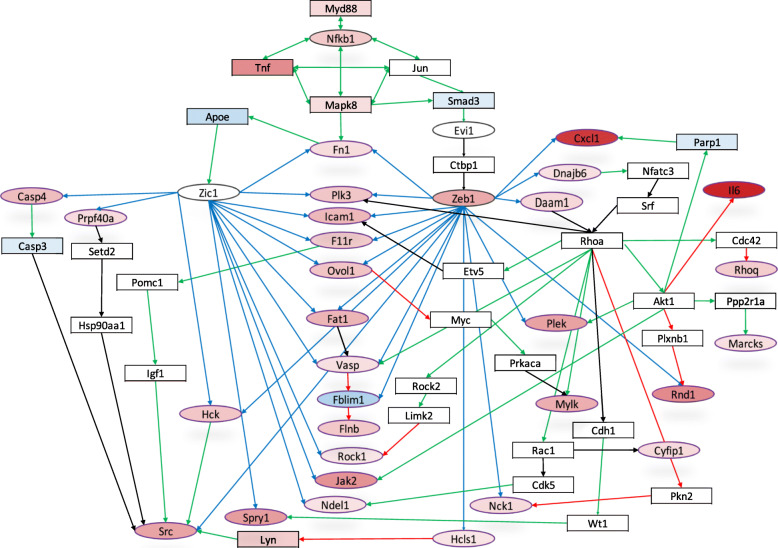
MyD88 is a master regulator upstream of two transcription factors with binding sites in the promotor regions of upregulated MyD88-dependent DEGs enriched in uptake processes. Ellipse nodes with black borders indicate transcription factors. MyD88, as a master regulator, is at the top of the network. Ellipse nodes with purple borders indicate genes that significantly enriched to uptake biological processes. The varying degree of red or blue hue in select nodes correlates with the gene’s Log2 Fold Change value. Red indicates positive fold change and blue indicates negative fold change. Gray nodes represent genes that were not differentially expressed. Green arrows indicate that the source node activates the target node. Red arrows indicate that the source node inhibits the target node. Black arrows indicate that the source node regulates the target node. Blue arrows are used to distinguish that the transcription factor source node has predicted binding sites in the promotor region of the target node

Based on our observation that the presence of MyD88 enhances phagocytosis (Fig. 1), we also analyzed whether any of the MyD88-dependent DEGs enriched to biological processes related to uptake. We identified 164 MyD88-dependent DEGs that enriched to five different biological processes relating to phagocytosis (Actin Filament Polymerization, Regulation of Cell Shape, Actin Cytoskeleton Organization, Cytoskeleton Organization, and Actin Filament Organization). Of particular interest, *Daam1* and *Fmnl1*, encoding two proteins known to play a role in phagocytosis of *Bb* [15, 37], were differentially expressed in an MyD88-dependent manner. Daam1, which was up-regulated, is a formin protein that bundles actin fibers together to increase stability of coiling pseudopods, which are more adept at capturing the highly motile spirochetes [68]. In contrast to *Daam1*, *Fmnl1* was down-regulated in response to *Bb*. Fmnl1 is also a formin protein that severs actin branches to promote polymerization and increase filopodia protrusion [68]. To determine whether MyD88 is a master regulator in any of these processes, we first identified transcription factors that map to promoter regions of the enriched DEGs. Analysis of these transcription factors revealed that Zic1 and Zeb1 have the capacity to bind to the promoter regions of several of the MyD88-dependent DEGs that significantly enriched to processes associated with bacterial uptake (Fig. 5). Zic1 is controlled by the intermediate protein ApoE, which is known to play a role in cholesterol metabolism in macrophages [69] and the absence of ApoE increases *Bb* burdens in experimentally infected mice [69]. We then used OCSANA, a specialized package available in Cytoscape [51] to link MyD88, as a master regulator, with transcription factors that map to DEGs in this specific subset. Based on this information we constructed a network illustrating potential links between MyD88-mediated signaling and up-regulated DEGs that may contribute to enhanced phagocytic capability seen in WT cells. The network (Fig. 5) shows *Rhoa*, *Akt1*, *Rac1* and *Cdc42* as genes that code for proteins which appear as intermediates on the network, meaning that their genes weren’t differentially expressed in our analysis. *Daam1* regulates *Rhoa* activity, which controls *Cdc42*, *Rac1* and *Akt1*. *Cdc42* activates a Rho GTPase, *Rhoq*, which is up-regulated in response to *Bb*. *Rac1* and *Akt1,* when translated*,* both activate multiple proteins whose corresponding genes are also up-regulated, indicating that while the genes for these intermediate proteins aren’t differentially expressed, they are still active in macrophages that have been stimulated with *Bb*. Taken together, these data suggest that MyD88 signaling upregulates multiple gene products involved in regulating macrophage membrane protrusions. Upregulation of these genes likely contributes to the reorganization of cell machinery that enhances the capability of the WT macrophage to take up spirochetes.

## Discussion

Previous studies by our group have emphasized that uptake and degradation of *Bb* by phagocytic cells, including monocytes and macrophages, are critical in eliciting the inflammatory response to the bacterium [14, 17–19, 29]. The findings from these studies, as well as others [28], show that the adaptor protein MyD88 plays a critical role in bacterial uptake and phagosomal signaling in macrophages. In the current study, we provide further evidence that the macrophage is a key driver of inflammation, even in the absence of MyD88. We also show that while MyD88 has a significant impact on spirochetal uptake, phagosome maturation and bacterial degradation are not affected (Fig. 1). Of particular novelty, phagosomal signaling cascades induced by *Bb* ligands in macrophages trigger a number of inflammatory and chemotactic pathways (Figs. 4 and S5). Moreover, the inflammatory processes are mediated by different regulatory proteins depending on whether MyD88 is present or absent, while induction of several chemotactic processes occurs independently of MyD88. In-depth analysis of these signaling cascades allowed us to identify previously underappreciated MyD88-dependent transcription factors which could lead to enhanced spirochetal uptake and clearance (Fig. 5).

To better understand the contribution of MyD88 to spirochete binding, uptake, degradation and signaling by macrophages, we used an ex vivo model. Murine macrophages lacking MyD88 show a phagocytic defect when stimulated with *Bb* ex vivo compared to WT macrophages. This defect in uptake has been previously demonstrated in macrophage stimulation experiments with other bacteria strains [31, 34, 35, 38]. Our results reveal that binding of *Bb* is not affected and that this phagocytic defect is not dependent on length of stimulation (i.e. time dependent) and is only slightly rescued by increasing the MOI. Thus, in the absence of MyD88, macrophages are still capable of binding and taking up the LD spirochete, but MyD88 signaling enhances the efficiency of *Bb* phagocytosis by macrophages. We also show here that when stimulated ex vivo, the macrophage response to *Bb* is driven by the signaling cascades induced by MyD88 as a result of bacterial ligands engaging TLR2 and TLR7 receptors in the phagosome (Figs. 2 and 4). Recognition in the phagosome is driven by degradation of bacteria, since more TLR2, TLR7 and MyD88 marker intensity were observed colocalizing with degraded spirochetes. Our results in Fig. 3 indicate that in the context of *Bb* infection, MyD88 is not required for phagosome maturation, evidenced by the recruitment of LAMP-1 to *Bb*-containing phagosomes in MyD88^−/−^ BMDMs. This is in contrast to Blander et al., who published that MyD88^−/−^ BMDMs infected with *Sa* or *E. coli* did not colocalize with either Lysotracker or LAMP-1 to the same degree as WT BMDMs [38]. One possible explanation for the different findings with our study is that the recruitment of LAMP-1 is delayed in MyD88^−/−^ BMDMs, given that Blander et al. measured phagosome maturation at an earlier time point than in our studies. This explanation is supported by Yates et al. who showed slightly delayed acidification in MyD88^−/−^ BMDMs stimulated with TLR2 or TLR4 ligands for 40 min [56]. The fragility of the *Bb* membranes also suggests that perhaps less acidification of the phagosome is needed to expose *Bb* PAMPs.

Reduced uptake of bacteria in macrophages lacking MyD88 is a phenotypic trait that has been extensively detailed [28, 31–35], but not well understood. To better understand the relationship between MyD88 signaling and phagocytosis, we used a computational systems biology approach. In prior studies, addition of TLR3 ligands to *Bb* stimulation of MyD88^−/−^ BMDMs significantly rescues uptake [36], suggesting that in the absence of MyD88 TRIF signaling can activate pathways that result in similar actin rearrangement in the cell. This signaling was shown to be mediated through PI3K [36], but interestingly PI3K was not differentially expressed in our macrophage stimulation. However, our network analysis from RNA-sequencing data identified DEGs that are up-regulated downstream of common phagocytosis effector proteins (Fig. 5). *Rhoq*, activated by *Cdc42* codes for TC10, a protein involved in generating long filopodia protrusions [70]. The gene *Cyfip1*, which encodes a part of the WAVE complex that regulates actin polymerization [71], was also up-regulated according to the network through *Rac1* protein interactions. The WAVE complex has higher involvement with lamellipodia formations [72]. It is likely that MyD88 controls transcription factors that upregulate these genes to promote phagocytosis through formation of coiling pseudopods, which are more similar to lamellipodia, rather than through straight filopodia protrusions. In addition to MyD88, studies indicating that TLR2 can utilize TRIF have also been completed, but this interaction only appears to contribute to the inflammatory response rather than spirochete uptake [18, 73]. More recently, the leukotriene LTB_4_ has been shown to promote phagocytosis of *Bb* by macrophages [74], but in our BMDM sequencing data we did not find differential expression of *Ltb4* or its receptor *Ltb4r1*. This could possibly be due to the later time point we selected for sequencing. It has also been shown that spleen tyrosine kinase (Syk) has an important role in phagocytosis of *Bb* via integrin binding [75]. The Syk gene (*Syk*) is significantly up-regulated in an MyD88-dependent manner, suggesting that MyD88 drives over-expression of *Syk* to increase phosphorylation and activation of proteins involved in generating actin branches. However, in our GO analysis *Syk* was not one of the 164 MyD88-dependent genes that enriched to uptake biological processes, and the transcription factor Zic1, which has binding sites in the promoter regions of a significant number of these genes, is not predicted to bind in the promoter region of *Syk*. Zic1 was of particular interest to us because it appeared downstream of MyD88 in our network analysis (Fig. 5) and is controlled by the intermediate protein ApoE. Mice lacking ApoE have increased bacterial burdens when infected with *Bb* [69], suggesting that ApoE signaling plays a role in cell remodeling processes necessary to enhance uptake. In addition, a link between *Bb* phagocytosis and cholesterol has been postulated by Hawley et al. who showed that CR3, a known phagocytic receptor for *Bb*, is recruited to lipid rafts with the co-receptor CD14 [76]. Thus, it is possible that MyD88 upregulates ApoE to enhance lipid rafts on the macrophage membrane, which can potentiate signaling to enhance uptake and provide scaffolding for proteins involved in actin remodeling.

Our computational analysis also supported that there are non-canonical sources of inflammation in MyD88^−/−^ mice. Our results suggest that there is possibly another receptor recruited to the phagosome that initiates chemokine production upon recognition of a *Bb* ligand. Another mechanism for triggering chemokine production may be that the TLR receptors are utilizing another adaptor protein to transmit signals out of the phagosome, as postulated by Petnicki-Ocwieja et al. [73]. Network analysis of DEGs from macrophages identified multiple master regulators that could be controlling production of these chemokines, but further investigation is needed to determine if these master regulators are in fact active in macrophages containing *Bb*. Additional studies to test whether acidification of the phagosome is required for *Bb*-induced chemokine production will also give insight into which ligand-receptor interaction induces this response.

## Conclusions

In summary, our results emphasize that the macrophage has a very important role in both recognition and clearance of *Bb* and is at the epicenter of the immunologic response to spirochete infection. The findings from these studies have also advanced our understanding of how phagosomal signaling drives spirochete uptake, recognition and inflammation. The adaptor protein MyD88 plays a critical role in these processes. Initial phagocytosis of *Bb* by macrophages does not require MyD88, but once taken up, recognition of *Bb* ligands exposed upon spirochete degradation occurs through endosomal TLRs which trigger MyD88-mediated signaling cascades. This signaling results in cell remodeling to enhance phagocytosis, as indicated by our ex vivo data, and allows macrophages to more efficiently internalize and clear the highly motile spirochetes by using more dynamic membrane protrusions. Further studies using the targets identified in these experiments may also provide insight into understanding the importance of phagocytosis in other bacterial infections. We can use similar techniques to look at the role of the macrophage response and MyD88 signaling in human macrophages, with the goal of increasing our understanding of the clinical spectrum associated with Lyme disease pathogenesis.

## Supplementary Information

**Additional file 1: Supplemental File 1.** Gene ontology analysis of differentially expressed genes.

**Additional file 2: Supplemental File 2.** Gene ontology analysis of identified transcription factors.

**Additional file 3: Supplemental File 3.** Gene ontology analysis of identified master regulators.

**Additional file 4: Supplemental File 4.** Identified differentially expressed genes.

**Additional file 5: Supplemental File 5.** Identified transcription factors.

**Additional file 6: Supplemental File 6.** Identified master regulators.

**Additional file 7: Figure S1.** Correlation of reads of RNA sequencing data. (A-D) Correlation of reads of RNA isolated from Bb-stimulated BMDMs. WT BMDMs were stimulated at an MOI 10:1 for 6 h (A). MyD88−/− BMDMs were stimulated at a MOI 100:1 for 6 h (B). To calculate differential expression, reads from the stimulated samples were normalized to unstimulated BMDMs of the same genotype; WT (C) or MyD88−/− (D).

**Additional file 8: Figure S2.** Secondary Control for TLR2. Confocal 40x images of internalized *Bb* with AF350 Secondary Antibody in WT BMDMs after stimulation at MOI 10:1. Green is *Bb*, blue is AF350 Secondary Antibody, and red is actin.

**Additional file 9: Figure S3.** MyD88^−/−^ BMDMs stimulated with *Bb* show abrogated cytokine production. (A-D) Quantification of IL-6 (A), TNFα (B), IL-10 (C) and CCL2 (D) proteins in supernatant from WT and MyD88^−/−^ (MyD) BMDMs stimulated with *Bb* at MOI 10:1 for 1, 4 or 6 h. *N* = 3 mouse BMDM per genotype *p-value< 0.05, **p-value< 0.01, ***p-value< 0.001, NS = not significant.

**Additional file 10: Figure S4.** *Bb* does not induce ASC formation in BMDMs. (A-D) Confocal 40x images (40x) of BMDMs stimulated with either *Sa* (A and C) or *Bb* (B and D) for 30 min (A-B) or 6 h (C-D). Blue is nucleus, red is ASC and green is the bacteria species.

**Additional file 11: Figure S5.** MyD88-privative MRs are significantly enriched in biological processes related to chemotaxis. (A) Venn diagram comparing biological processes (BP) relating to chemotaxis significantly enriched between MyD88-dependent (light gray) and MyD88-privative (dark gray) master regulators. (B) Heat map showing fold change of master regulators enriched in chemotaxis biological processes in MyD88^−/−^ (yellow) BMDMs. GO numbers for significantly enriched BP are indicated on the x-axis.

**Additional file 12.**

**Additional file 13.**

**Additional file 14.**

**Additional file 15.**

**Additional file 16.**

## Data Availability

Catalog numbers and suppliers for commercially available materials used in these studies are provided. Non-commercially available materials can be provided upon request. ImageJ software can be downloaded at no cost from nih.gov. Cytoscape can be downloaded at no cost from cytoscape.org. GeneXplain is a subscription software that can be purchased from genexplain.com.

## Abbreviations

LD: Lyme disease
*Bb*: *Borrelia burgdorferi*
TLR: Toll-like receptor
PAMPs: Pathogen associated molecular patterns
WT: Wild type
UCH: UConn Health
GFP: Green fluorescent protein
BSK: Barbour Stoner Kelly
*Sa*: *Staphylococcus aureus*
BMDM: Bone marrow derived macrophages
BSA: Bovine serum albumin
DEG: Differentially expressed genes
GE: Gene Ontology
MOI: Multiplicities of infection
ASC: Apoptosis-associated speck-like protein containing a CARD
