# Supplementary figures and images for: Macrophage mediated recognition and clearance of Borrelia burgdorferi elicits MyD88-dependent and -independent phagosomal signals that contribute to phagocytosis and inflammation
Source: BMC Immunol. 2021 May 17;22:32. doi: 10.1186/s12865-021-00418-8 (PMC8127205; doi:10.1186/s12865-021-00418-8)

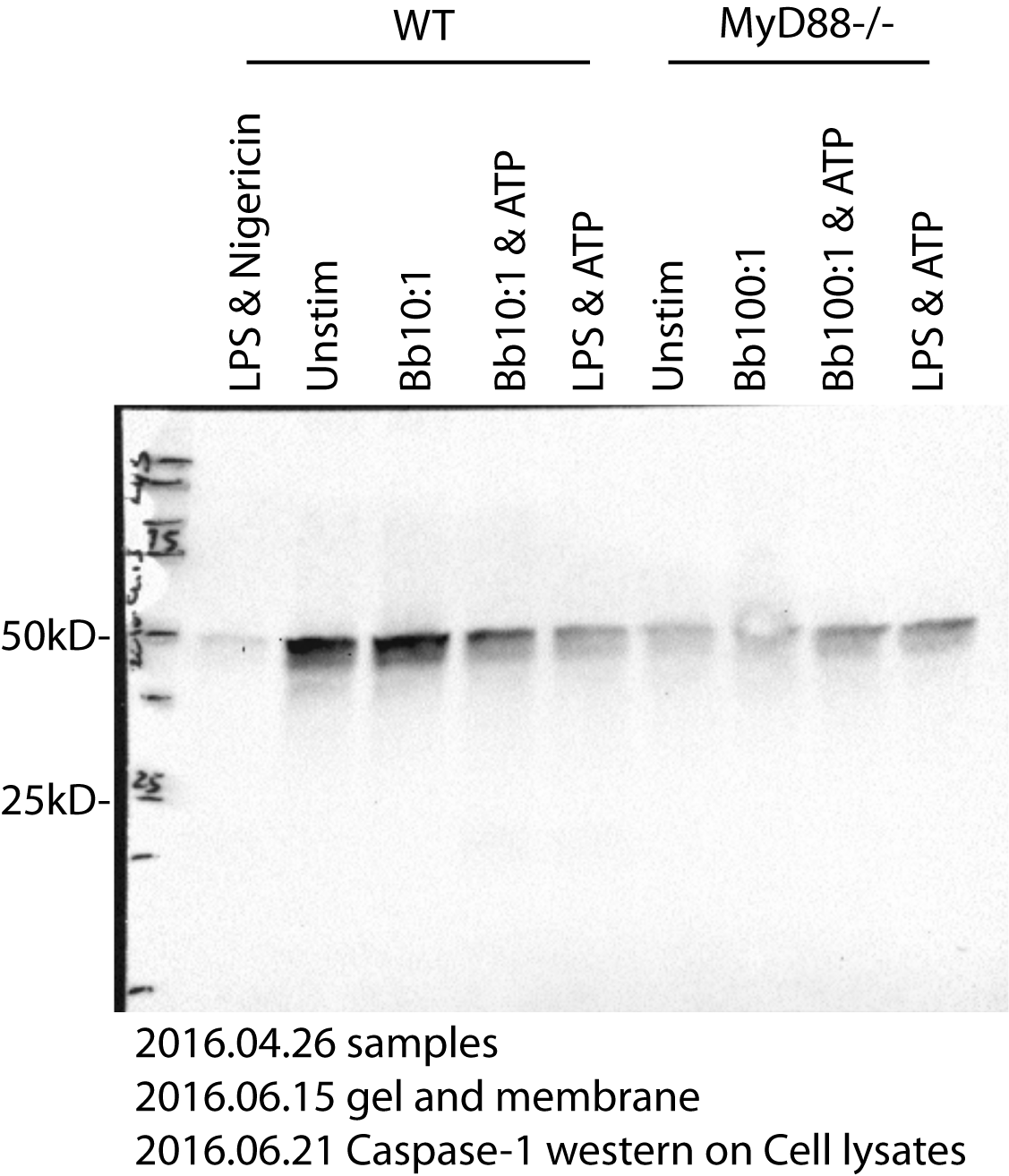

Supplement: Supplementary file 12 — Additional file 12. [file 12865_2021_418_MOESM12_ESM.tif]

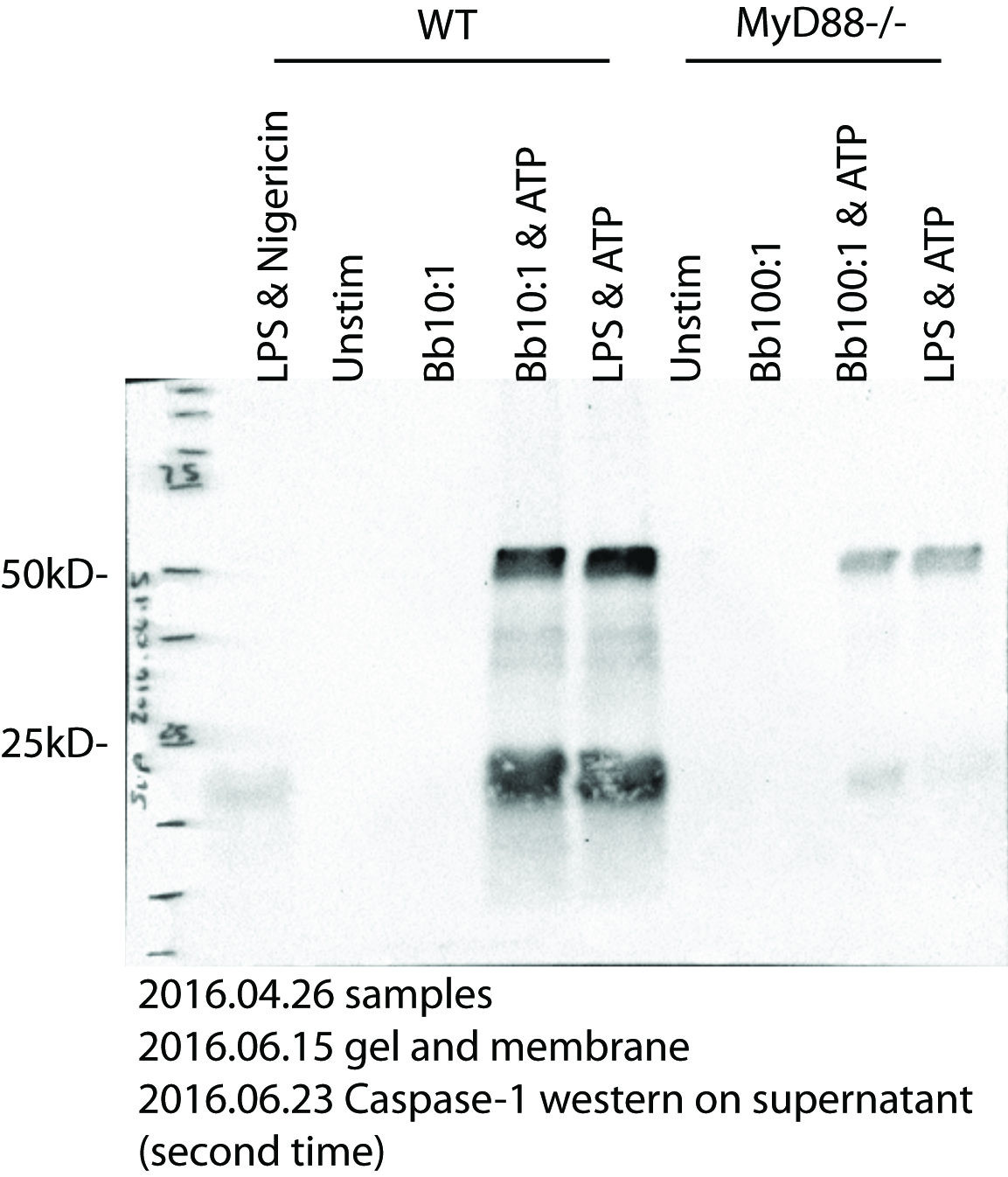

Supplement: Supplementary file 13 — Additional file 13. [file 12865_2021_418_MOESM13_ESM.tif]

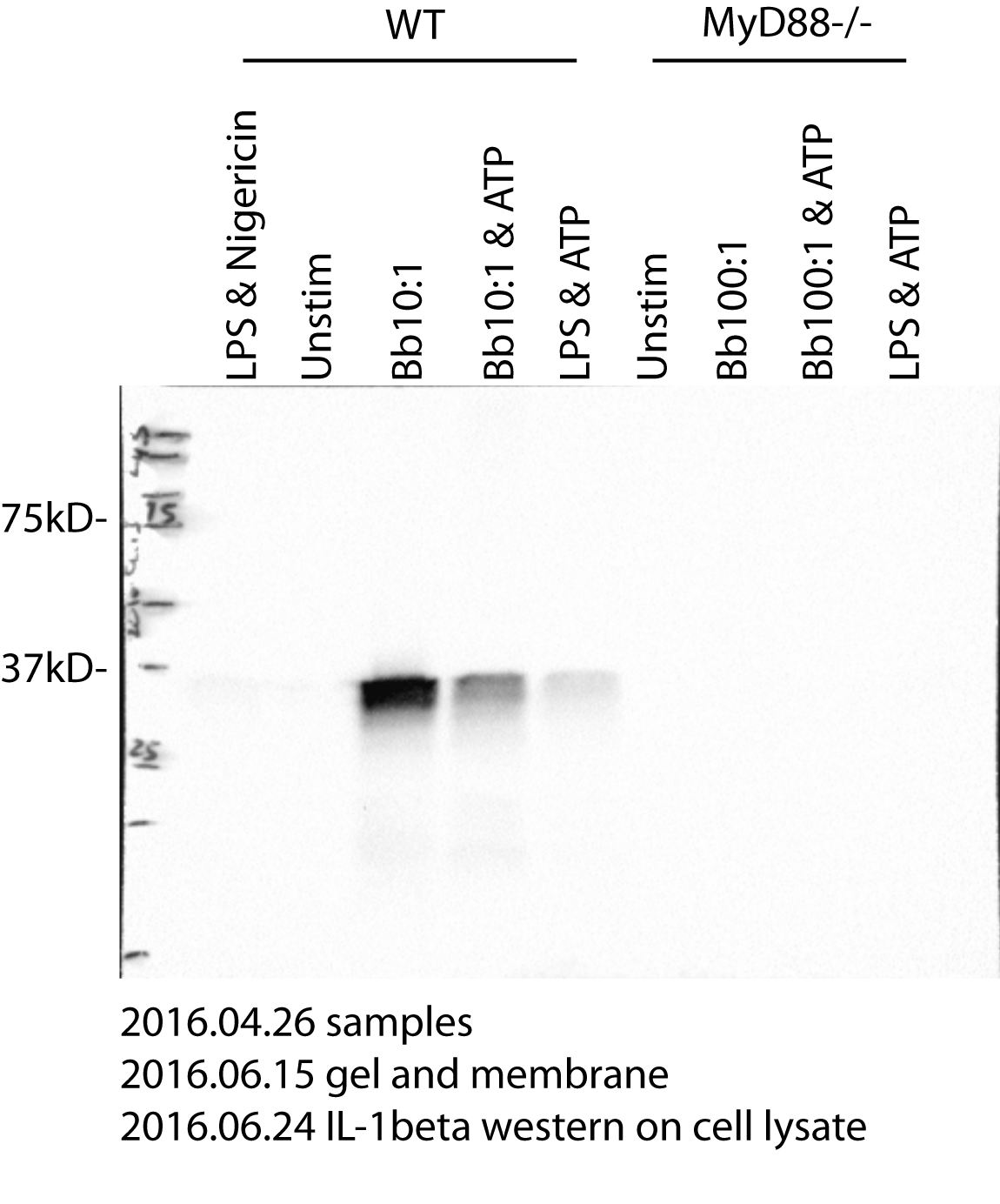

Supplement: Supplementary file 14 — Additional file 14. [file 12865_2021_418_MOESM14_ESM.tif]

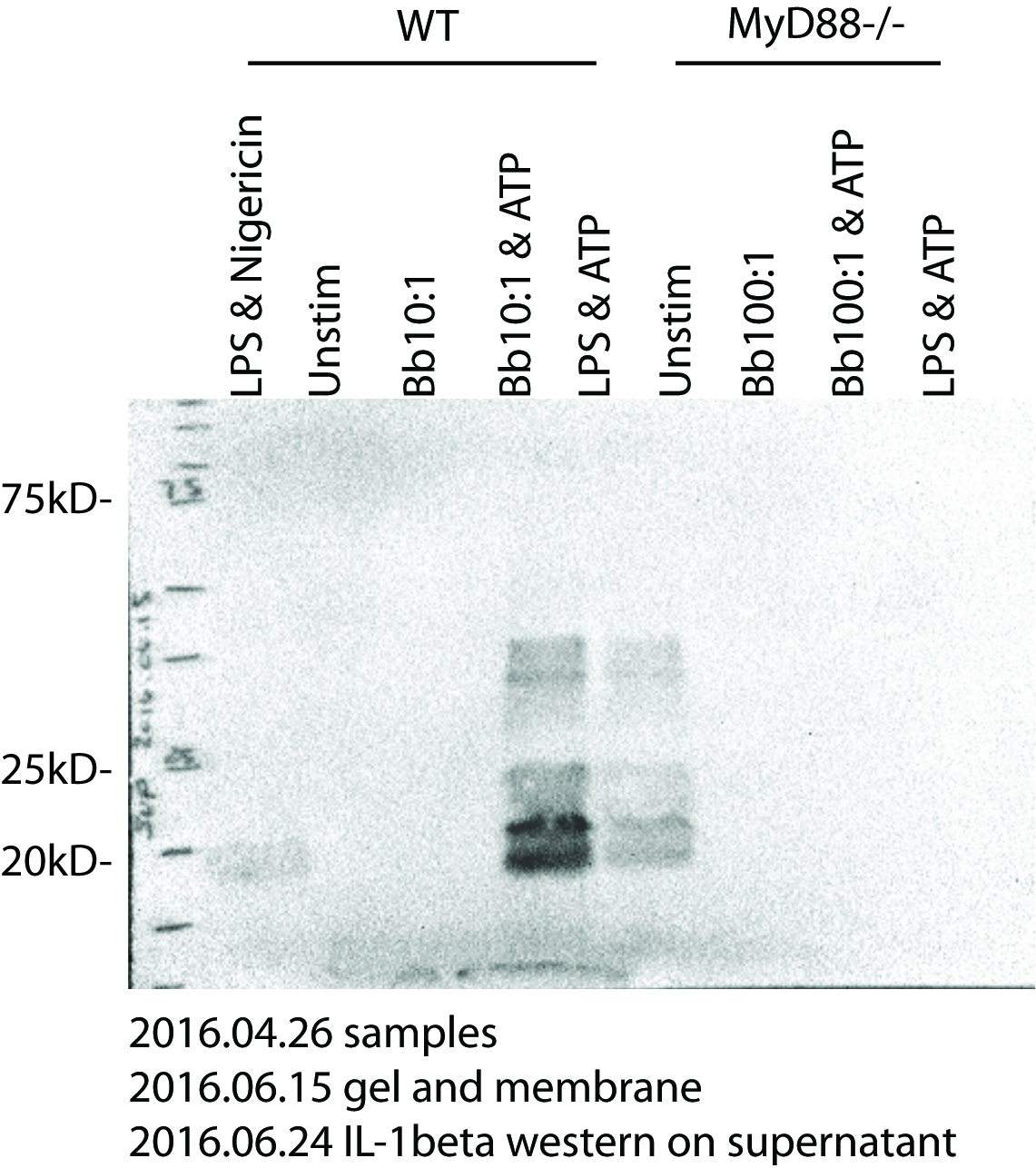

Supplement: Supplementary file 15 — Additional file 15. [file 12865_2021_418_MOESM15_ESM.tif]

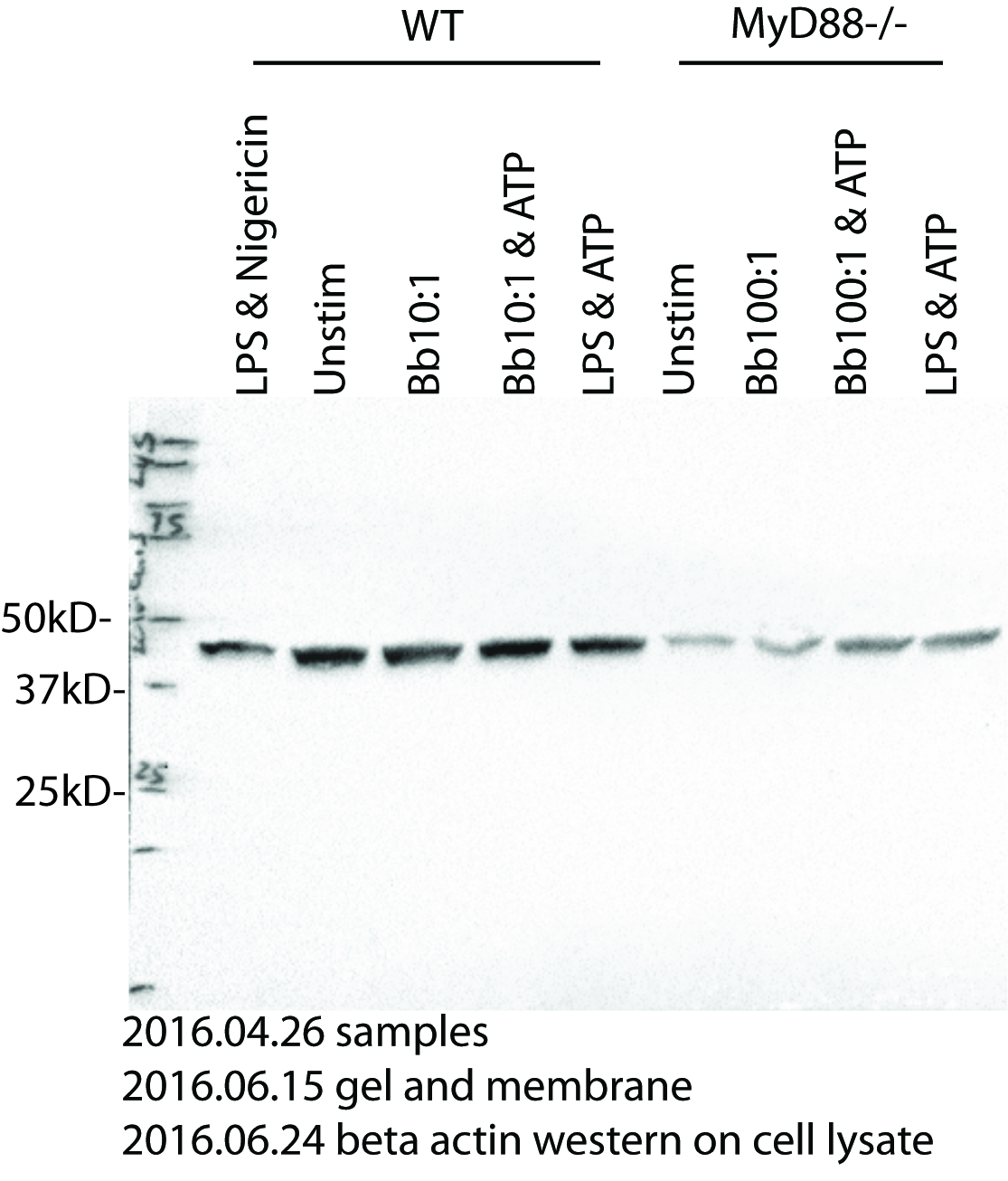

Supplement: Supplementary file 16 — Additional file 16. [file 12865_2021_418_MOESM16_ESM.tif]
